# Training set design in genomic prediction with multiple biparental families

**DOI:** 10.1002/tpg2.20124

**Published:** 2021-07-24

**Authors:** Xintian Zhu, Willmar L. Leiser, Volker Hahn, Tobias Würschum

**Affiliations:** ^1^ State Plant Breeding Institute Univ. of Hohenheim Stuttgart 70593 Germany; ^2^ Institute of Plant Breeding, Seed Science and Population Genetics Univ. of Hohenheim Stuttgart 70593 Germany

## Abstract

Genomic selection is a powerful tool to reduce the cycle length and enhance the genetic gain of complex traits in plant breeding. However, questions remain about the optimum design and composition of the training set. In this study, we used 944 soybean [*Glycine max* (L.) Merr.] recombinant inbred lines from eight families derived through a partial–diallel mating design among five parental lines. The cross‐validated prediction accuracies for the six traits seed yield, 1,000‐seed weight, protein yield, plant height, protein content, and oil content were high, ranging from 0.79 to 0.87. We investigated among‐family predictions, making use of the special mating design with different degrees of relatedness among families. Generally, the prediction accuracy decreased from full‐sibs to half‐sib families to unrelated families. However, half‐sib and unrelated families also showed substantial variation in their prediction accuracy for a given family, which appeared to be caused at least in part by the shared segregation of quantitative trait loci in both the training and prediction sets. Combining several half‐sib families in composite training sets generally led to an increase in the prediction accuracy compared with the best family alone. The prediction accuracy increased with the size of the training set, but for comparable prediction accuracy, substantially more half‐sibs were required than full‐sibs. Collectively, our results highlight the potential of genomic selection for soybean breeding and, in a broader context, illustrate the importance of the targeted design of the training set.

## Core Ideas

- A partial–diallel design was used to optimize the training set for genomic predictions
- The prediction accuracy decreased from full‐sibs to half‐sibs to unrelated families
- The allelic state of major QTL in the training & prediction sets affected prediction accuracy
- Composite training sets are recommended

**Abbreviations**
P1: Parent 1: Gallec
P2: Parent 2: Primus
P3: Parent 3: Protina
P4: Parent 4: Sultana
P5: Parent 5: Sigalia
QTL: quantitative trait loci
RIL: recombinant inbred line
RR‐BLUP: Ridge regression best linear unbiased prediction

## 1 INTRODUCTION

Genomic selection was first suggested for animal breeding and has been shown to enhance the rate of genetic improvement (Hayes, Bowman, Chamberlain, & Goddard, 2009; Meuwissen et al., 2001). Genomic selection has also emerged as a valuable tool for plant breeding. It refers to the selection of individuals on the basis of the genomic estimated breeding value derived by estimating the effects of markers across the genome and using them for prediction. In contrast to marker‐assisted selection on identified medium‐ to large‐effect quantitative trait loci (QTL), this approach also captures the effects of small‐effect loci, making it particularly appealing for genetically complex traits. Different models for genomic selection have been proposed, which differ in how they model the genetic architecture of the traits to be predicted. Ridge regression best linear unbiased prediction (RR‐BLUP) assumes that the entire genome contributes to the trait and thus an effect is estimated for all markers. Ridge regression best linear unbiased prediction has been shown to be a reliable method with a high prediction accuracy across crops and traits. An extension of this model is weighted RR‐BLUP, which allows the incorporation of known major QTL as fixed effects while all other markers are treated as random effects (Bernardo, 2014; Zhang et al., 2014; Spindel et al., 2016; Herter et al., 2019; Rice & Lipka, 2019).

Soybean [*Glycine max* (L.) Merr.] is among the most important food and feed crops worldwide. Its seeds contain approximately 40% protein, which makes soybean the most important protein source for animal feed globally (Patil et al., 2017; Van & McHale, 2017). Soybean seeds also contain around 20% oil and most of the cultivars grown worldwide are primarily bred for high oil content (Sato et al., 2014). In Europe, soybean is not only used for livestock, but with the trend towards vegetarian or vegan lifestyles, soy‐based products like the traditional Asian food tofu have become increasingly popular (Kurasch, Hahn, et al., 2018; Kurasch, Leiser, et al., 2018). Because of the strong dependency of Europe on protein imports as well as the increasing global demand for soybean, early‐maturing cultivars adapted to higher latitudes have been bred, and soybean cultivation has been expanded in Central and Northern Europe in recent years (Hahn & Würschum, 2014; Würschum et al., 2019).

Genomic selection has been evaluated for a range of crops, including sugar beet (*Beta vulgaris* L.) (Würschum et al., 2013), oat (*Avena sativa* L.) (Asoro et al., 2011), rye (*Secale cereale* L.) (Wang et al., 2014), rapeseed (*Brassica napus* L.) (Würschum et al., 2014; Zou et al., 2016), barley (*Hordeum vulgare* L.) (Schmidt et al., 2016; Thorwarth et al., 2017), maize (*Zea mays* L.) (Zhao et al., 2012; Pace et al., 2015; Li et al., 2019), triticale (× *Triticosecale* Wittmack) (Würschum et al., 2017), and wheat (*Triticum aestivum* L.) (He et al., 2016; Huang et al., 2016; Lozada et al., 2020). For soybean, comparably few studies are available, although the approach has the potential to assist breeding for disease resistance (Bao et al., 2014; Wen et al., 2018; Ravelombola et al., 2019), abiotic stress resistance (Jähne et al., 2019), and yield or yield‐related agronomic traits (Jarquín et al., 2014; Ma et al., 2016; Zhang et al., 2016; Duhnen et al., 2017; Matei et al., 2018; Đorđević et al., 2019; Stewart‐Brown et al., 2019; Ravelombola et al., 2020).

The accuracy of genomic prediction is defined as the correlation between the estimated and the true breeding values, and determines the efficiency and thus the potential of the application of this approach in selection (i.e., in genomic selection). This prediction accuracy has been shown to depend on different factors, such as training set size and marker density, as well as linkage disequilibrium or co‐segregation between markers and QTL so that the effects of the QTL can be captured for prediction (Habier, Fernando, & Dekkers, 2007; Habier, Fernando, & Garrick, 2013; Daetwyler, Villanueva, et al., 2008; Daetwyler, Pong‐Wong, et al., 2010; Schopp, Müller, Technow, et al., 2017). Importantly, however, it also uses genetic relationships between individuals in the training and in the prediction set, and RR‐BLUP is well suited to exploit this kinship information.

**Core Ideas**

- A partial–diallel design was used to optimize the training set for genomic predictions
- The prediction accuracy decreased from full‐sibs to half‐sibs to unrelated families
- The allelic state of major QTL in the training & prediction sets affected prediction accuracy
- Composite training sets are recommended

For genomic selection to be applied, a training set composed of individuals with both genotypic and phenotypic data is required to estimate the marker effects. On the basis of these, we can predict the performance of genotyped but untested individuals in the prediction set. In plant breeding programs, new crosses are constantly being made, and the aim is to apply genomic prediction to identify the best‐performing progeny in the resulting segregating families (Würschum, 2012). Genomic prediction generally works best within biparental families (i.e., for full‐sibs), which, however, would require to be some individuals from that family being phenotyped before genomic selection can be applied to the remaining lines. Alternatively, lines with existing phenotypic and genotypic data can be chosen for the training set. These will typically also be from biparental families and can be half‐sibs to the lines in the family to be predicted, if they share a common parental line, or can be from unrelated families. Prediction accuracy has been shown to depend substantially on the genetic relatedness between the training set, and the prediction set and decreases with decreasing genetic relatedness between them, even producing negative prediction accuracies for unrelated families (Riedelsheimer et al., 2013; Lehermeier et al., 2014; Schopp, Müller, Technow, et al., 2017; Schopp, Müller, Wientjes, et al., 2017; Würschum et al., 2017; Brauner et al., 2020). An alternative to using single families is to combine individuals from different families to form the training set, which raises questions about the optimal design of such composite training sets (Brauner et al., 2020).

The aim of this study was to evaluate the accuracy of genomic prediction within and across families in a large partial–diallel with 944 soybean recombinant inbred lines (RILs) derived from eight families genotyped with 10,893 molecular markers. The special mating design has the advantage that, for each family, individuals with varying degrees of relatedness are available, which enabled us to investigate the effect on the prediction accuracy and thus on the optimal composition of the training set in genomic prediction. In particular, our objectives were to (a) explore the genomic prediction accuracy for the six traits seed yield, 1,000‐seed weight, protein yield, plant height, protein content, and oil content; (b) test the performance of RR‐BLUP among a series of models and compare the cross‐validated prediction accuracy between a full random model and a model incorporating major QTL as fixed effects; (c) perform reciprocal predictions for any pair of families and assess the prediction accuracy for single families with a model trained either with all other families, with half‐sib families, or with unrelated families; (d) analyze the prediction accuracy of composite training sets consisting of half‐sib families or unrelated families; and (e) evaluate the effect of training set size on the prediction accuracy for models trained with a full‐sib family, with half‐sib families, or with unrelated families.

## 2 MATERIALS AND METHODS

### 2.1 Plant materials

This study was based on a total of 944 F_5:8_ RILs from eight families derived from a partial–diallel mating design with five parents, developed by single seed descent (Kurasch et al., 2017). Five varieties that showed good agronomic performance in Central Europe were used as parents: Parent 1, ‘Gallec’ (P1); Parent 2, ‘Primus’ (P2); Parent 3, ‘Protina’ (P3); Parent 4, ‘Sultana’ (P4); Parent 5 ‘Sigalia’ (P5). The eight families and their family size are as follows: P1 × P2 (*n* = 104), P1 × P3 (*n* = 117), P1 × P5 (*n* = 234), P2 × P5 (*n* = 80), P2 × P3 (*n* = 117), P2 × P4 (*n* = 106), P3 × P5 (*n* = 94), and P4 × P5 (*n* = 92).

### 2.2 Phenotypic data

The field experiments and the phenotypic evaluation of the six agronomic traits (seed yield, 1,000‐seed weight, protein yield, plant height, protein content, and oil content) have been described in our previous study (Kurasch et al., 2017). In brief, all RILs were divided into three trials according to their maturity date in the year before the experiment, including early (Trial 1), mid‐early (Trial 2), and late types (Trial 3). The five parents were grown in all three trials; additionally, 38 (16 from P1 × P5, 12 from P2 × P5, and 10 from P3 × P5) and 66 (14 from P1 × P2, three from P1 × P3, seven from P1 × P5, 14 from P2 × P3, 17 from P2 × P4, five from P2 × P5, and six from P3 × P5) overlapping RILs were chosen to connect Trial 1 with Trial 2 and Trial 2 with Trial 3, respectively. In 2014, the field experiments were performed at three locations in Germany, with plot sizes of 9 m^2^ (1.5 by 6 m) and 65 seeds m^−2^, conducted with a partially replicated design with 20% of the lines grown in a replication at each location. The replicated lines differed among the three locations, so that 60% of the lines were replicated across the three trials. Seeds were harvested and dried to an equal moisture content, then used to evaluate seed yield and 1,000‐seed weight. Plant height was measured as the distance between the ground and the last trifoliate leaf at the beginning of maturity. Seed protein content and oil content were assessed by near‐infrared spectroscopy with a Polytec 2120 spectrometer (Polytec GmbH). Best linear unbiased estimates across locations and trials were calculated for all genotypes as described by Kurasch et al. (2017) and used for subsequent analyses. Notably, the model included a trial effect nested within location to adjust for differences among the three trials grouped according to the RILs’ maturity.

### 2.3 Genomic prediction

All soybean lines were genotyped by genotyping‐by‐sequencing and a consensus map was constructed as described previously (Zhu et al., 2020). The total number of markers in the consensus map was 10,893, with an average map distance of 0.89 cM across all 20 chromosomes. Genomic selection was performed by RR‐BLUP (Endelman, 2011). The prediction accuracy was determined through 1,000 runs of fivefold cross‐validation for the following three scenarios. In Scenario 1, 80% of the lines from each family were randomly sampled and then combined as the training set. The remaining 20% of the lines from each family formed the prediction set, either combined across families or separately for each family. The second scenario involved within‐family prediction, in which, for each family, 80% individuals were sampled at random as the training set and the remaining 20% were used for prediction. Scenario 3 involved across‐family prediction with one family as the prediction set and different combinations of half‐sib families or unrelated families forming the training set. In addition, based on our results from QTL and association mapping (Zhu et al., 2020, 2021), identified major QTL were included as fixed effects in a weighted RR‐BLUP in the first two scenarios described above. The prediction accuracy was calculated as *r*_GS _= *r*_MP_/*h* (Lande & Thompson, 1990; Zhao et al., 2012), where *r*_MP_ indicates the Pearson's correlation between the estimated best linear unbiased estimates and the genomic estimated breeding values, and *h* is the square root of the heritability.

In order to test the suitability of RR‐BLUP, we also chose a series of Bayesian models implemented in the R package BGLR (Pérez & De Los Campos, 2014), including Bayesian ridge regression, BayesA (Meuwissen et al., 2001), BayesB (Habier et al., 2011), BayesC (Habier et al., 2011), Bayesian LASSO (Park & Casella, 2008), and Bayesian reproducing kernel Hilbert space regression (Wahba, 1990). The comparison was made only for the whole dataset and because of the time‐consuming nature of these models, we changed some parameters (nIter = 20,000; burnIn = 4,000; thin = 5) and set the prior mode of the residual variance to match 20% phenotypic variance (*R* ^2^ = 0.8).

## 3 RESULTS

### 3.1 The large diallel design and comparison of prediction models

**FIGURE 1:**
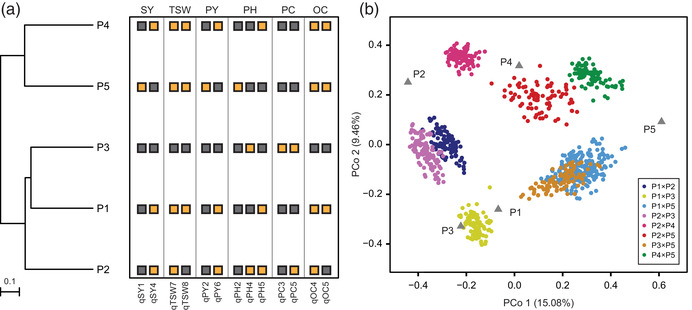
Genetic characterization of the parental lines and the recombinant inbred lines (RILs). (a) Clusterplot showing the genetic relationship among the five parental lines and their allelic state at major quantitative trait loci (QTL) identified for the six traits. Orange indicates the favorable allele. SY, seed yield; TSW, 1,000‐seed weight; PY, protein yield; PH, plant height; PC, protein content; OC, oil content. (b) Principal coordinate (PCo) plot of the five parental lines and the 944 RILs derived from eight families. P1, Parent 1 (Gallec); P2, Parent 2 (Primus); P3, Parent 3 (Protina); P4, Parent 4 (Sultana); P5, Parent 5 (Sigalia)

The population underlying this study consisted of 944 RILs derived from eight families (Figure 1a) (Kurasch et al., 2017). The size of the eight families varied from 80 to 234 individuals, and because of the partial–diallel mating design with five parental lines, each family had at least four half‐sib families and one unrelated family (Figure 1b, Supplemental Figure S1). The estimated heritabilities of the six evaluated traits were 0.77 for seed yield, 0.94 for 1,000‐seed weight, 0.70 for protein yield, 0.91 for plant height, 0.85 for protein content, and 0.87 for oil content (Kurasch et al., 2017). This dataset was thus ideally suited to dissect the effect of different degrees of relatedness between the training and prediction sets on the accuracy of genomic prediction.

**FIGURE 2:**
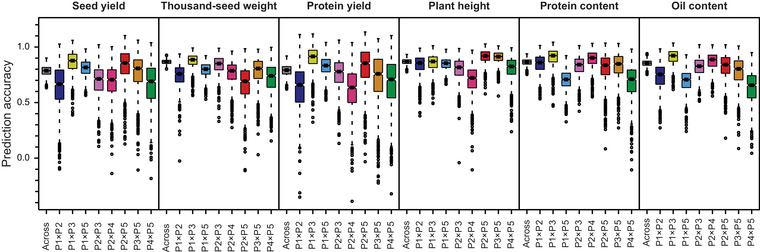
Prediction accuracy across families and for each of the eight families assessed by fivefold cross‐validation with a full random model trained across all families. P1, Parent 1 (Gallec); P2, Parent 2 (Primus); P3, Parent 3 (Protina); P4, Parent 4 (Sultana); P5, Parent 5 (Sigalia)

We first compared the performance of RR‐BLUP and different Bayesian models (Bayesian ridge regression, BayesA, BayesB, BayesC, Bayesian LASSO, and Bayesian reproducing kernel Hilbert space regression) via fivefold cross‐validation in the whole population. Our results showed that the prediction accuracy of RR‐BLUP was always among the best for all six investigated traits; consequently, RR‐BLUP was used for all subsequent analyses (Supplemental Figure S2, Supplemental Table S1). We started by assessing the genomic prediction accuracy across families and for each single family, via fivefold cross‐validation with 1,000 runs and a full random model trained with 80% of the progeny from each of the eight families (Figure 2). The median accuracy of cross‐family prediction was 0.79 for seed yield, 0.87 for 1,000‐seed weight, 0.79 for protein yield, 0.87 for plant height, 0.87 for protein content, and 0.86 for oil content. With the same training set, within‐family predictions generally yielded prediction accuracies comparable with those obtained across families for all six traits, but prediction in individual families resulted in a much larger variation of the prediction accuracy than the predictions across families. However, the prediction set sizes were only 16 to 23 (the remaining 20% of lines from the respective family), except for the largest family (P1 × P5), for which the prediction set size was 47 and which showed the lowest variation in prediction accuracy. In addition to a full random model, we chose the medium‐ or large‐effect QTL associated with the six traits and included them as fixed effects in the model (Figure 1a) (Zhu et al., 2020, 2021). However, we did not observe an apparent increase in prediction accuracy across families or within most of the single families (Supplemental Figure S3).

### 3.2 Reciprocal prediction among families

**FIGURE 3:**
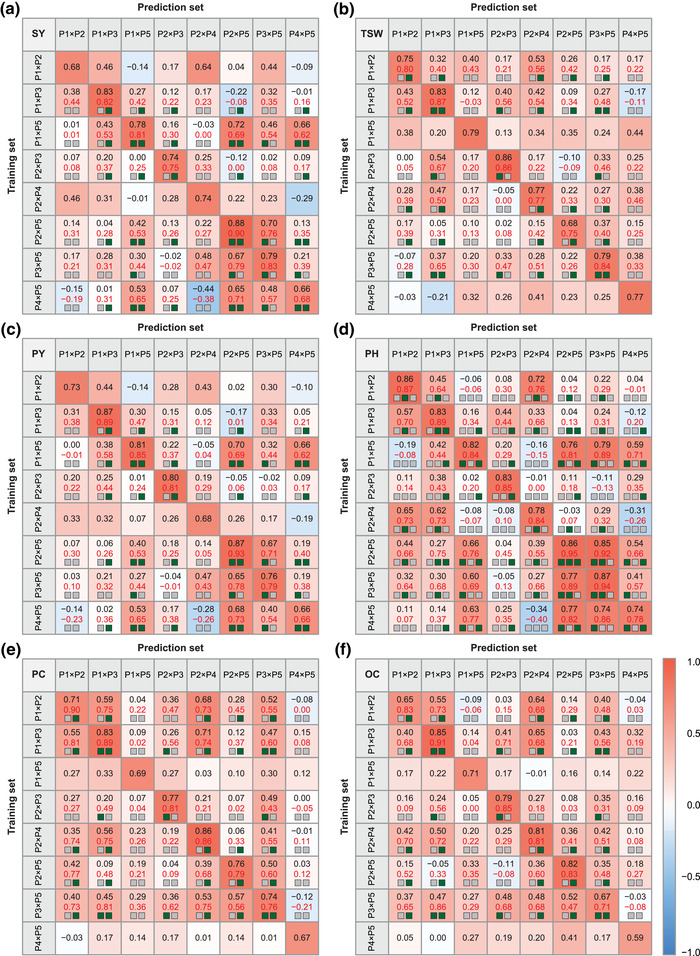
Reciprocal predictions among families. The diagonal shows the median of the cross‐validation prediction accuracy within each family. The off‐diagonal elements show the prediction accuracy for each family observed when another family was used as the training set. Black indicates the prediction accuracies of a full random model; red indicates those obtained by a model incorporating the major quantiatative trait loci (QTL) as fixed effects, provided that the QTL are polymorphic in the training set family. The boxes refer to the QTL shown in Figure 1, in which gray indicates segregation of the QTL in the training set but not in the prediction set and green shows segregation in both the training and prediction sets. (a) SY, seed yield; (b) TSW, 1,000‐seed weight; (c) PY, protein yield; (d) PH, plant height; (e) PC, protein content; (f) OC, oil content. P1, Parent 1 (Gallec); P2, Parent 2 (Primus); P3, Parent 3 (Protina); P4, Parent 4 (Sultana); P5, Parent 5 (Sigalia)

Next, we estimated the prediction accuracy among the eight families, with one family being used as the training set and another for prediction, thereby testing all reciprocal combinations. The prediction accuracy assessed by cross‐validation within each family can serve as a reference for these among‐family predictions, as this within‐family prediction scenario, where the training and prediction sets consisted of full‐sibs, generally yielded the best prediction accuracy (Figure 3, Supplemental Table S2). The prediction accuracies for a single family varied substantially with the different families being used as the training set, even negative prediction accuracies occurred. In general, we found that having half‐sib families as the training set resulted in better prediction accuracy than the use of unrelated families. Reciprocal predictions between families generally yielded prediction accuracies of comparable magnitude but, in part, also revealed substantial differences. We again tested the model incorporating QTL as fixed effects if these QTL segregated in the training set family used for estimating the effects. Notably, these QTL do not have to segregate in the family to be predicted. Compared with the full random model, the prediction accuracy generally improved if one or even two of the QTL also segregated in the prediction set family (Figure 3). This difference depended on the trait, the family, and the segregating QTL. For protein and oil content, the effect of including major QTL as fixed effects in the prediction model was most pronounced, as the prediction accuracy often increased strongly and sometimes more than doubled.

### 3.3 Factors affecting the prediction accuracy

**FIGURE 4:**
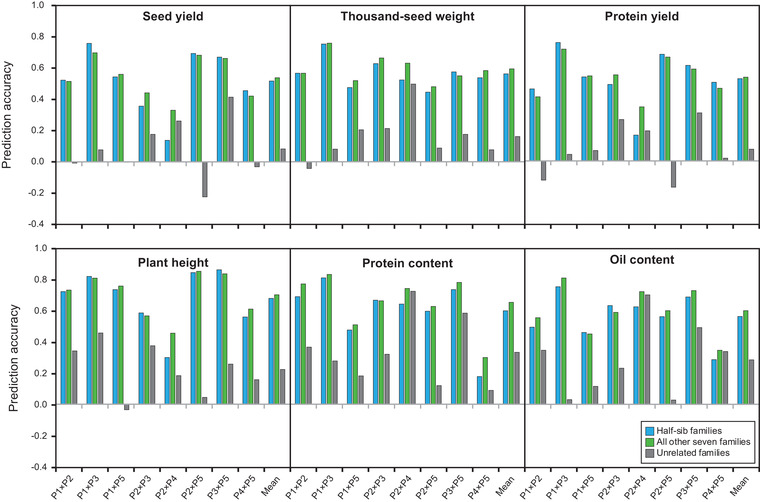
Prediction accuracy for each family assessed by a full random model with either half‐sib families, all the other families, or unrelated families as the training set. In addition, the means across all families are shown. P1, Parent 1 (Gallec); P2, Parent 2 (Primus); P3, Parent 3 (Protina); P4, Parent 4 (Sultana); P5, Parent 5 (Sigalia)

The among‐family predictions showed the dependency of the prediction accuracy on the relatedness of the individuals in the training and prediction sets. We therefore investigated the genomic prediction accuracy for each family with a full random model trained by half‐sib families, all of the other seven families, or unrelated families (Figure 4). The predictions based on half‐sib families and all other families both resulted in similar high prediction accuracies. Predictions based on unrelated families as the training set, by contrast, resulted in low prediction accuracy. A notable exception was the prediction of protein and oil content in the P2 × P4 family, for which the unrelated families achieved a prediction accuracy as high as that of the half‐sib families.

**FIGURE 5:**
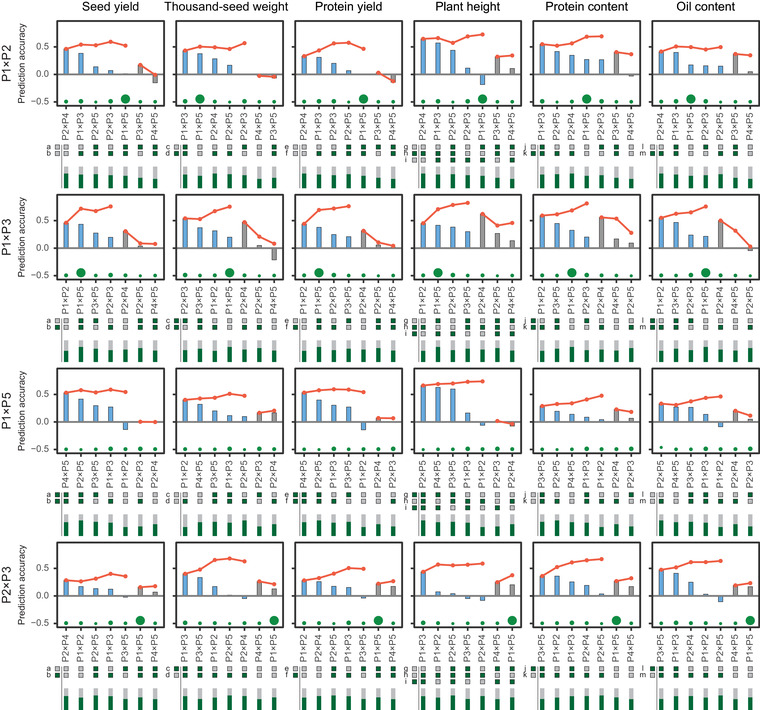
Genomic prediction accuracy of a single family based on single‐family or composite training sets. P1, Parent 1 (Gallec); P2, Parent 2 (Primus); P3, Parent 3 (Protina); P4, Parent 4 (Sultana); P5, Parent 5 (Sigalia). The results are shown for four families (P1 × P2, P1 × P3, P1 × P5, and P2 × P3) that were predicted from their half‐sib families (blue) or unrelated families (gray), as well as composite training sets (red). For the latter, half‐sib families and unrelated families were ranked in descending order of their prediction accuracy for the respective family and sequentially added to the training set. The green dots show the sample size of each family. The squares refer to the allelic state of the major quantitative trait loci (QTL) in each family, and the two leftmost squares represent the family to be predicted, with green indicating segregation of the QTL. QTL: a, qSY1; b, qSY4; c, qTSW7; d, qTSW8; e, qPY2; f, qPY6; g, qPH2; h, qPH4; i, qPH5; j, qPC3; k, qPC5; l, qOC4; m, qOC5. The bar plots indicate the proportion of shared segregating markers (green) within the total markers in the family to be predicted (i.e., the proportion of markers in that family for which an estimated effect was available for prediction)

From this result, the question arose as to whether predictions based on half‐sib families generally have a high prediction accuracy and, conversely, unrelated families a low one, or if optimized training sets can be designed. Therefore, we next addressed the variation in prediction accuracy obtained with individual half‐sib or unrelated families, and the effect of combining them in training sets (Figure 5, Supplemental Figure S4). The half‐sib families as well as the unrelated families were ordered according to their prediction accuracy for the family to be predicted. This revealed substantial variation in prediction accuracy of the half‐sib families, with some being high but some often being rather low or even negative. Likewise, the unrelated families also showed this variation in their prediction accuracy, with generally lower ones but some unrelated families having a higher prediction accuracy than that achieved with some half‐sib families. This variation in prediction accuracy did not appear to be related to the size of the family used for effect estimation or to the proportion of markers in the family to be predicted for which an effect was estimated (i.e., which also segregated in the training set family).

We then investigated whether segregation of the medium‐ to large‐effect QTL in the training and prediction set families affected the prediction accuracy. This is best exemplified by protein and oil content, for which a major QTL on chromosome 20 (qPC5/qOC5) was previously identified that explains approximately 50% of the genotypic variance in the families in which it segregates (Zhu et al., 2020). Generally, the prediction accuracy was higher if this QTL segregated in the family to be predicted. Particularly in the families P1 × P5 and P4 × P5, in which none of the two QTL segregated, the prediction accuracy was low, even for half‐sib families. Interestingly, if this QTL segregated in the training set family, prediction set families that also segregated for the QTL generally achieved a higher prediction accuracy. For example, for the family P1 × P2, the three half‐sib families that also segregated for this QTL had a higher prediction accuracy than the two half‐sib families that did not segregate for this QTL. The same held true for the unrelated families; in our example, the unrelated family P3 × P5 achieved a higher prediction accuracy than the two half‐sib families that did not segregate for the QTL. There were, of course, a few exceptions where a half‐sib or unrelated family segregated for the QTL but nevertheless had a low prediction accuracy (e.g., for the prediction of the family P1 × P3 by the unrelated family P2 × P5). Another clear example for the effect of QTL segregation in both the training and prediction sets is plant height, for which a major QTL (qPH2) was identified on chromosome 6 (Zhu et al., 2021). Prediction set families that segregated for this QTL had high prediction accuracies if the training set family also segregated for this QTL.

### 3.4 Assessing composite training sets

In addition to predictions based on single half‐sib or unrelated families, we evaluated the prediction accuracy of composite training sets where the half‐sib or unrelated families were added to their training sets sequentially in the order of their prediction accuracy (Figure 5, Supplemental Figure S4). For example, for seed yield prediction in P1 × P2, P2 × P4 had the highest prediction accuracy and formed the first training set, then P1 × P3 with the second highest prediction accuracy was added to the training set, then P2 × P5 with the third highest prediction accuracy, and so on. Thus, the training set sizes became larger but families with lower prediction accuracies on their own were added. When half‐sib families were added to the composite training set, we observed a slight increase or a plateau in the prediction accuracy. Exceptions were the predictions in P2 × P4 for seed yield, protein yield, and plant height and in P4 × P5 for seed yield and protein yield, for which the prediction accuracy decreased, often because of the low or strong negative prediction accuracy of single families. In contrast, for the unrelated families, the prediction accuracy of the composite training sets often plateaued at a low level or decreased.

### 3.5 Variations in training set size

**FIGURE 6:**
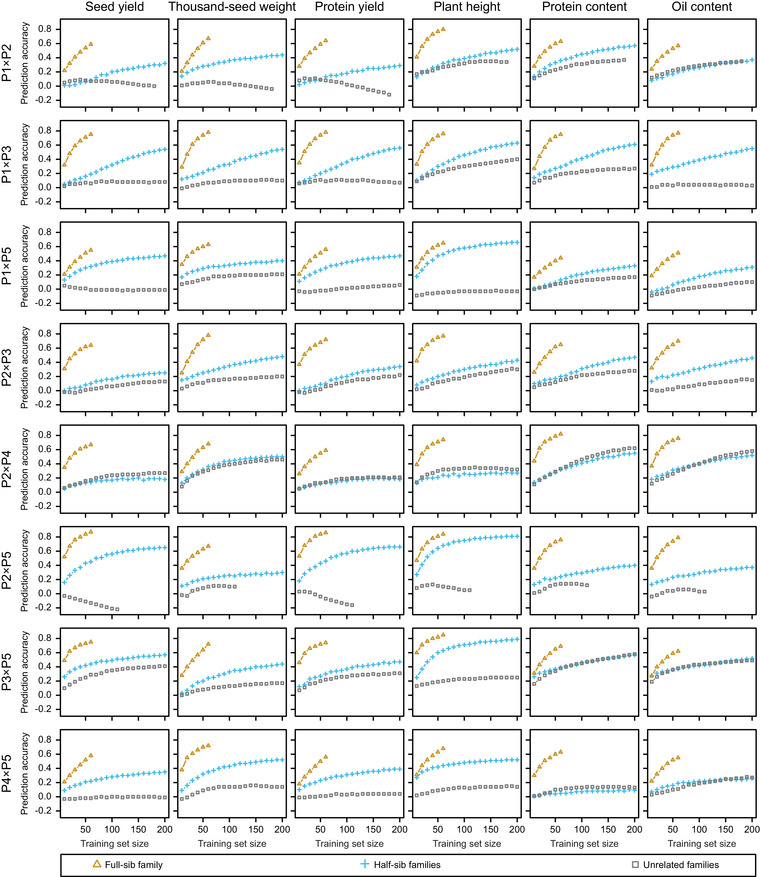
Dependency of the prediction accuracy on the training set size. Median genomic prediction accuracy of each family assessed by fivefold cross‐validation with 1,000 runs for different sizes of training set comprised of either full‐sibs, half‐sib families, or unrelated families. P1, Parent 1 (Gallec); P2, Parent 2 (Primus); P3, Parent 3 (Protina); P4, Parent 4 (Sultana); P5, Parent 5 (Sigalia)

Last, we used a resampling strategy to evaluate the effect of different training set sizes for predictions based on full‐sibs within one family, half‐sib families, or unrelated families (Figure 6). For full‐sibs and half‐sibs, the prediction accuracy increased continuously with an increasing training set size. An exception was the prediction accuracy based on half‐sib families for plant height in the families P1 × P5, P2 × P5, P3 × P5, and P4 × P5, which showed a comparably strong initial increase in the families segregating for the major QTL qPH2. For full‐sib families, even a moderate training set size (e.g., 30–40 lines) achieved reasonable prediction accuracies for most traits and families. For half‐sibs, by contrast, a similar number of lines yielded only low prediction accuracies usually ranging between 0.0 and 0.2, and many more individuals than with full‐sibs were required to achieve a similar prediction accuracy. For unrelated families, the prediction accuracy also increased with increasing training set size, but more slowly. However, for some families and traits, it also plateaued at a very low level or even decreased, thereby resembling the results observed for the composite training sets.

## 4 DISCUSSION

Genomic selection has been shown to be a valuable tool to assist the improvement of complex traits in plant breeding. Though it is known that the degree of relatedness between training and prediction sets is an important parameter affecting the prediction accuracy, the optimal composition of the training set remains a challenge. In this study, we used a large diallel design with 944 soybean RILs to address this issue, making use of the different degrees of relatedness among families offered by this special mating design. In addition, our aim was to provide a comprehensive assessment of the potential of using genomic selection for important agronomic traits in soybean breeding populations.

### 4.1 Choice of the prediction model and incorporation of QTL

Different genomic prediction models have been developed and evaluated, among which RR‐BLUP has emerged as a very robust, reliable and efficient method that has therefore been widely used in animal and plant breeding (Heffner et al., 2009; Heslot et al., 2012), and has also been applied in soybean (Ma et al., 2016; Zhang et al., 2016; Ravelombola et al., 2019; Stewart‐Brown et al., 2019). In this study, we compared seven different models, which substantiated the use of RR‐BLUP because, in agreement with previous reports, we observed highly similar prediction accuracies among the models for all traits (Supplemental Table S1, Supplemental Figure S2) (Bao et al., 2014; Đorđević et al., 2019; Ravelombola et al., 2019).

A characteristic of RR‐BLUP is that it may underestimate the effects of major QTL (Bernardo, 2014). Consequently, previous studies incorporated selected QTL as fixed effects in the genomic prediction model, which often resulted in higher prediction accuracies (Boeven et al., 2016; Spindel et al., 2016; Würschum et al., 2018). By contrast, in a simulation study, it was more often observed that the inclusion of fixed‐effect QTL had a negative effect on the prediction accuracy of 216 different simulated genetic architectures (Rice & Lipka, 2019). Here, however, we did not observe an apparent improvement in the genomic prediction accuracy for any of the six traits in the entire panel (Supplemental Figure S3). This may be because these previous studies were based on diversity panels, whereas biparental families with large linkage blocks were used here. In combination with high‐density molecular markers, as used in our study, the effects of QTL can be captured by RR‐BLUP if they are distributed across enough markers linked to the QTL. Alternatively, the potential positive effects of including QTL in the prediction model may have been obscured in this prediction across several families that only partly segregated for each QTL and which may also have varied for the effects of the QTL because of their different genetic backgrounds. In predictions from one family to another, incorporating QTL improved the prediction accuracy in several cases, especially for traits like protein content, oil content, and plant height, for which single QTL explain a rather large proportion of the genotypic variance (Figure 3, Supplemental Table S2). Thus, the potential benefit of including QTL as fixed effects depends on the genetic architecture of the target trait. If major QTL are known, there is no disadvantage to include them as fixed effects in the model, as the prediction accuracy was not adversely affected by their inclusion.

### 4.2 Genomic prediction accuracies in soybean

The prediction accuracies for the six investigated traits (seed yield, 1,000‐seed weight, protein yield, plant height, protein content, and oil content), assessed by cross‐validation across all families, were generally high, ranging from 0.79 for seed yield and protein yield to 0.87 for plant height, 1,000‐seed weight, and protein content. This supports previous findings from different crops, showing that the higher the genetic complexity of the trait, the lower the prediction accuracy (Würschum et al., 2014, 2017; Zhang et al., 2015; Michel et al., 2016; Herter et al., 2019). For soybean, Ma et al. (2016) also evaluated genomic predictions for plant height and seed yield based on 235 soybean varieties grown at multiple locations, and reached prediction accuracies of 0.86 and 0.47, respectively. It must be noted that different results were obtained for yield with different breeding materials, with prediction accuracies ranging from 0.47 to 0.75 (Jarquín et al., 2014; Ma et al., 2016; Xavier et al., 2016; Duhnen et al., 2017; Matei et al., 2018; Stewart‐Brown et al., 2019). Collectively, however, these results demonstrate the potential of genomic selection to assist soybean breeding for complex traits.

Use of a training set across families yielded different medians and variations in the prediction accuracy for the eight families (Figure 2). Although this is partly caused by the smaller prediction set sizes of some families (e.g., P2 × P5: 16 individuals; P4 × P5: 18 individuals), it also shows that a certain level of variation in the prediction accuracy must be expected, which underlines the importance of ensuring the optimal composition of the training set.

### 4.3 Prediction accuracy within and among families

We evaluated reciprocal predictions among all pairs of families and compared these prediction accuracies with those obtained by cross‐validation within individual families (Figure 3, Supplemental Table S2). The different prediction accuracies found when predicting either way between two families may have been caused by different family sizes or a distinct genetic architecture of the trait in each family (Würschum et al., 2017; Herter et al., 2019). In general, the prediction accuracies obtained within a family with full‐sibs for training and prediction yielded the highest prediction accuracies, followed by prediction based on half‐sib families, whereas unrelated families resulted in the lowest prediction accuracies. These results confirmed the strong effect of relatedness between the training and prediction sets on the prediction accuracy (Habier et al., 2007, 2010; Hayes, Bowman, Chamberlain, Verbyla, et al., 2009; Riedelsheimer et al., 2013; Schopp, Müller, Wientjes, et al., 2017; Brauner et al., 2020). Notably, however, although the above conclusion holds true in general, there was substantial variation in the prediction accuracy achieved by different half‐sib families, as well as by unrelated families (Figure 5, Supplemental Figure S4). Thus, for a single family, not every half‐sib family resulted in a high prediction accuracy. For some families and traits, most half‐sib families performed well; sometimes, they showed a continuous decrease from the one with the highest to the one with the lowest prediction accuracy. In some cases, only one or two showed high prediction accuracies, whereas the others had very low or even negative prediction accuracies. The suitability of a half‐sib family for predicting a given family generally showed the same pattern across all six traits, so if a half‐sib family achieved a low, medium, or high prediction accuracy relative to the other half‐sib families, it usually did so consistently for all traits.

This was unlikely to be caused by differences in training set size, as all families ranged between 80 and 117 individuals except for one with 234, which, however, was also often among the medium or poor performing training sets. In addition, we investigated if these differences might have been caused by the proportion of markers in the family to be predicted for which an effect estimate was available (i.e., that also segregated in the training set family). These usually showed small differences that did not appear to be related to the differences in prediction accuracy. Although some criteria have been suggested to distinguish the highly and poorly predictive families to be included in a training set (Lehermeier et al., 2014; Schopp, Müller, Wientjes, et al., 2017), Brauner et al. (2020) investigated various quantitative genetic parameters and concluded that genomic information alone is insufficient to determine the expected prediction accuracy and thus the optimal training set composition. Notably, the expected kinship between several half‐sib families was identical, but the parental lines had different relatedness; thus, molecularly, some half‐sib families are closer to a given family than others, which may have partly contributed to the observed differences (Figure 1, Supplemental Figure S1).

In addition, the different prediction accuracies realized by different half‐sib families may reflect the similarity in the genetic architecture, such as the proportion of common QTL segregating in the families used for training and prediction. Moreover, it may depend on the QTL effects, as these may vary with the genetic background and thus between half‐sib families. Schopp, Müller, Wientjes, et al. (2017) performed a simulation study and concluded that QTL that account for a sizeable proportion of the genotypic variance in the prediction set can result in low prediction accuracy if they do not segregate in the training set. Although this is readily conceivable, it is difficult to prove in experimental datasets. Genomic prediction is applied to complex traits as it can also capture the effects of small‐effect QTL, which, however, are so numerous and small in effect that pinpointing the effect of any one of them on the prediction accuracy is futile. In our diallel design, we had previously identified QTL for all six traits (Zhu et al., 2020, 2021). A major QTL for protein and oil content that explains approximately half of the genotypic variance in the families in which it segregates provided the opportunity to investigate the effect of shared QTL between the training and prediction sets on the prediction accuracy. Our results indicate that if this QTL segregates in both sets, the prediction accuracy increases. Though this may appear obvious, it suggests that the same will hold true for medium‐ and small‐effect QTL. Thus, an important factor that determines the prediction accuracy is whether the QTL segregating in the prediction set also segregate in the training set.

### 4.4 Genomic prediction with composite training sets

Our results on the effect of the degree of relatedness on the prediction accuracy, as well as the effect of variation among half‐sib families used as the training set, strongly suggest the use of composite training sets as opposed to single families. We predicted six traits in single families with three composite groups: all other families, half‐sib families, or unrelated families (Figure 4). In most cases, we observed that predictions of a single family based on all seven families or based on its half‐sib families yielded comparable and medium to high prediction accuracy, whereas unrelated families alone resulted in low prediction accuracy. Thus, adding unrelated families to the training set did not decrease the prediction accuracy, which is most probably because there were always more half‐sib families than unrelated families. Having an excess of unrelated families in a training set will probably not be compensated for any more by having relatively fewer half‐sib families, as suggested by the findings from a simulation study (Brauner et al., 2020). The composite training sets consisting of only half‐sib families added sequentially according to their prediction accuracy mostly showed a weak continuous increase; only in very few exceptions was the prediction accuracy of all half‐sib families combined lower than that of the best half‐sib family. Notably, however, in a breeding program, the best half‐sib family for predicting the target family would not be known. These results thus corroborate previous findings from a simulation study that found a stable increase in the prediction accuracy with sequential addition of the ranked half‐sib families to the composite training set (Brauner et al., 2020).

In conclusion, our results suggest that if several half‐sib families are available, these should be combined to form the training set. In cases where not enough such material is available to provide the required size of the training set, other related material should be used. This might be selected on the basis of pedigree and/or molecular information. For traits for which major QTL have been identified, the training set should preferentially segregate for these QTL, as the example of protein and oil content illustrated that unrelated families can also achieve high prediction accuracy. In general, however, unrelated families should be excluded from the training set, given their expectedly low or even negative prediction accuracies, and the consequent reduction of the prediction accuracy of the composite training set.

### 4.5 Effect of training population size on prediction accuracy

Previous studies have shown that the size of the training population is a crucial factor affecting the accuracy of genomic prediction (Zhao et al., 2012; Jarquín et al., 2014; Crossa et al., 2017; Stewart‐Brown et al., 2019). We therefore also investigated this in training sets made up of different relatedness groups (i.e., full‐sib families, half‐sib families, and unrelated families) (Figure 6). For full‐sib families, the prediction accuracy showed a continuous increase, which appeared to be steeper up to approximately 40 individuals, then increased more slowly. For the half‐sib families, a more variable picture was observed, depending on the family and the trait. Often, there was also a steeper increase for 50 to 100 individuals; thereafter, the increase in prediction accuracy slowed or even plateaued. For those showing a continuous increase, 200 individuals achieved approximately the same prediction accuracy as 20 full‐sibs. This is in line with the findings from Lehermeier et al. (2014), who reported that in maize, 375 half‐sib lines reached the same prediction accuracy as 50 full‐sibs. This illustrates that similar prediction accuracies can be obtained with half‐sibs as with full‐sibs, but only with much larger training set sizes.

Regarding unrelated families, the increase in prediction accuracy with training set size was slowest, also starting to plateau after about 100 individuals or even earlier. The few examples with decreasing prediction accuracy (e.g., seed yield in P1 × P2 and P2 × P5) were found in training sets composed of only one or two families, with always one family with negative prediction accuracy (Figure 5, Supplemental Figure S4). If only a few individuals from the families with negative prediction accuracy are sampled into the training set, this results in a prediction accuracy of around zero, but with more individuals added, the prediction accuracy decreases (Figure 6). Taken together, our results underline the importance of the size of the training set in genomic prediction and emphasize that this is highly dependent on the relatedness between the training and prediction sets.

### 4.6 Conclusions for the application of genomic selection in breeding

Generally, the ideal composition of the training set in genomic selection remains challenging, but following the above guidelines should increase the chance of achieving satisfactory prediction accuracies and thus the expected selection gain (Supplemental Figure S5). Which type of material is available as training set also depends on the crop and the organization of the breeding program. If some individuals from a family have already been phenotyped, then the remaining individuals are best predicted by these full‐sibs, provided a minimum number of full‐sibs is available (Marulanda et al., 2015). If the number of full‐sibs is too low, they can be combined with half‐sib families (Brauner et al., 2020). If, by contrast, all individuals from a family are to be predicted to select those that should enter an intensive field evaluation, the best option is to go for half‐sibs or similarly related material. Knowing the power of half‐sib families for prediction, a breeding program incorporating genomic selection, could try to ensure that for any new cross that is initiated and for which the aim is to apply genomic selection to the progeny, enough half‐sib families are available to form a reliable and effective composite training set. The use of unrelated families appears generally risky and their inclusion in a composite training set may only have a positive effect if the training set is otherwise too small and if they share a similar genetic architecture. In summary, our results illustrate the potential of genomic selection for soybean breeding and, in a broader context, substantiate some general rules about the optimization of genomic selection in plant breeding.

## CONFLICT OF INTEREST

The authors declare that they have no conflict of interest.

## AUTHOR CONTRIBUTIONS

Xintian Zhu: data curation, formal analysis, visualization, writing—original draft. Willmar Leiser: data curation, writing—review and editing. Volker Hahn: conceptualization, data curation, funding acquisition, writing—review and editing. Tobias Würschum: conceptualization, funding acquisition, writing—review and editing.

## Supporting information

Zhu_et_al_SupplementaryMaterial.PDF
**Supplemental Table S1** Median of prediction accuracy across all eight families assessed by fivefold cross‐validation via RR‐BLUP and different Bayesian models (20,000 iterations and 4,000 burn‐in).
**Supplemental Table S2** Results of reciprocal prediction among the families from Figure 3.
**Supplemental Figure S1** Genetic relatedness among the 944 RILs shown as a kinship matrix, based on 10,893 markers.
**Supplemental Figure S2** Comparison of the genomic prediction accuracy across all eight families assessed by fivefold cross‐validation with different models.
**Supplemental Figure S3** Prediction accuracy across families and for each of the eight families assessed by fivefold cross‐validation via a model with major QTL included as fixed effects and trained across all eight families.
**Supplemental Figure S4** Genomic prediction accuracy of a single family based on single families or composite training sets.
**Supplemental Figure S5** General guidelines on the optimization of genomic prediction in plant breeding. TS, training set; PS, prediction set.

Zhu_et_al_Data and code.xlsx
Supplemental File S1 Data and code.
