# Supplementary material for: Training set design in genomic prediction with multiple biparental families
Source: Plant Genome. 2021 Jul 24;14(3):e20124. doi: 10.1002/tpg2.20124 (PMC12807416; doi:10.1002/tpg2.20124)
Supplement: Supplementary file 1 [file TPG2-14-e20124-s002.pdf]

Training set design in genomic prediction with multiple biparental families

Xintian Zhu[1], Willmar L. Leiser[1], Volker Hahn[1], Tobias Würschum[2,*]

[1]State Plant Breeding Institute, University of Hohenheim, 70593 Stuttgart, Germany

[2] Institute of Plant Breeding, Seed Science and Population Genetics, University of Hohenheim, 70593 Stuttgart, Germany

✉ Tobias Würschum, email: tobias.wuerschum@uni-hohenheim.de

Supplementary material

TABLE S1 Median of prediction accuracy across all eight families assessed by fivefold cross-validation using RR-BLUP and different Bayesian models (20,000 iterations and 4,000 burn-in).

	Seed yield	Thousand-seed weight	Protein yield	Plant height	Protein content	Oil content
RR-BLUP	0.79	0.87	0.79	0.87	0.87	0.86
BRR	0.79	0.87	0.79	0.87	0.87	0.86
BayesA	0.79	0.87	0.80	0.88	0.88	0.87
BayesB	0.80	0.88	0.80	0.88	0.88	0.87
BayesC	0.79	0.87	0.80	0.87	0.88	0.86
BL	0.79	0.87	0.79	0.87	0.87	0.86
RKHS	0.79	0.87	0.79	0.87	0.87	0.86

RR-BLUP: ridged regression best linear unbiased prediction; BRR: Bayesian Ridge Regression; BL: Bayesian LASSO; RKHS: Bayesian Reproducing Kernel Hilbert Spaces Regression.

TABLE S2 Result of reciprocal prediction among families from Figure 3. The prediction accuracies of a full random model are shown in black, that of a model incorporating major QTL as fixed effect are shown in red underneath.

Trait[a]			PS[c]							
			P1×P2	P1×P3	P1×P5	P2×P3	P2×P4	P2×P5	P3×P5	P4×P5
SY	TS[b]	P1×P2	0.68	0.46	−0.14	0.17	0.64	0.04	0.44	−0.09
		P1×P3	0.38	0.83	0.27	0.12	0.17	−0.22	0.32	−0.01
			(0.44)	(0.82)	(0.42)	(0.22)	(0.23)	(−0.08)	(0.35)	(0.16)
		P1×P5	0.01	0.43	0.78	0.16	−0.03	0.72	0.46	0.66
			(0.01)	(0.53)	(0.81)	(0.30)	(0.00)	(0.69)	(0.54)	(0.62)
		P2×P3	0.07	0.20	0.00	0.74	0.25	−0.12	0.02	0.09
			(0.08)	(0.37)	(0.25)	(0.75)	(0.33)	(0.00)	(0.08)	(0.17)
		P2×P4	0.46	0.31	−0.01	0.28	0.74	0.22	0.23	−0.29
		P2×P5	0.14	0.04	0.42	0.13	0.22	0.88	0.70	0.13
			(0.31)	(0.28)	(0.53)	(0.26)	(0.27)	(0.90)	(0.76)	(0.35)
		P3×P5	0.17	0.28	0.30	−0.02	0.48	0.67	0.79	0.21
			(0.21)	(0.31)	(0.44)	(−0.02)	(0.47)	(0.79)	(0.83)	(0.39)
		P4×P5	−0.15	0.01	0.53	0.07	−0.44	0.65	0.48	0.66
			(−0.19)	(0.31)	(0.65)	(0.25)	(−0.38)	(0.71)	(0.57)	(0.68)

			PS							
			P1×P2	P1×P3	P1×P5	P2×P3	P2×P4	P2×P5	P3×P5	P4×P5
TSW	TS	P1×P2	0.75	0.32	0.40	0.17	0.53	0.26	0.17	0.17
			(0.80)	(0.40)	(0.43)	(0.21)	(0.56)	(0.42)	(0.25)	(0.22)
		P1×P3	0.43	0.83	0.12	0.40	0.42	0.09	0.27	−0.17
			(0.52)	(0.87)	(−0.03)	(0.56)	(0.54)	(0.34)	(0.48)	(−0.11)
		P1×P5	0.38	0.20	0.79	0.13	0.34	0.35	0.24	0.44
		P2×P3	0.00	0.54	0.17	0.86	0.17	−0.10	0.33	0.25
			(0.05)	(0.67)	(0.20)	(0.86)	(0.22)	(−0.09)	(0.46)	(0.22)
		P2×P4	0.28	0.47	0.17	−0.05	0.77	0.22	0.27	0.38
			(0.39)	(0.50)	(0.23)	(0.00)	(0.77)	(0.33)	(0.30)	(0.46)
		P2×P5	0.17	0.05	0.10	0.02	0.15	0.68	0.37	0.15
			(0.39)	(0.31)	(0.13)	(0.08)	(0.42)	(0.75)	(0.40)	(0.25)
		P3×P5	−0.07	0.37	0.20	0.33	0.28	0.22	0.79	0.38
			(0.28)	(0.65)	(0.30)	(0.47)	(0.51)	(0.26)	(0.84)	(0.33)
		P4×P5	−0.03	−0.21	0.32	0.26	0.41	0.23	0.25	0.77

			PS							
			P1×P2	P1×P3	P1×P5	P2×P3	P2×P4	P2×P5	P3×P5	P4×P5
PY	TS	P1×P2	0.73	0.44	−0.14	0.28	0.43	0.02	0.30	−0.10
		P1×P3	0.31	0.87	0.30	0.15	0.05	−0.17	0.33	0.05
			(0.38)	(0.89)	(0.47)	(0.31)	(0.12)	(0.01)	(0.34)	(0.21)
		P1×P5	0.00	0.38	0.81	0.22	−0.05	0.70	0.32	0.66
			(−0.01)	(0.58)	(0.85)	(0.37)	(0.04)	(0.69)	(0.44)	(0.62)
		P2×P3	0.20	0.25	0.01	0.80	0.19	−0.05	−0.02	0.09
			(0.22)	(0.44)	(0.24)	(0.81)	(0.29)	(0.06)	(0.03)	(0.17)
		P2×P4	0.33	0.32	0.07	0.26	0.68	0.26	0.17	−0.19
		P2×P5	0.07	0.06	0.40	0.18	0.14	0.87	0.67	0.19
			(0.30)	(0.26)	(0.53)	(0.25)	(0.05)	(0.93)	(0.71)	(0.40)
		P3×P5	0.03	0.21	0.27	−0.04	0.47	0.65	0.76	0.19
			(0.10)	(0.32)	(0.44)	(−0.01)	(0.43)	(0.78)	(0.79)	(0.38)
		P4×P5	−0.14	0.02	0.53	0.17	−0.28	0.68	0.40	0.66
			(−0.23)	(0.36)	(0.65)	(0.38)	(−0.26)	(0.73)	(0.54)	(0.66)

			PS							
			P1×P2	P1×P3	P1×P5	P2×P3	P2×P4	P2×P5	P3×P5	P4×P5
PH	TS	P1×P2	0.86 (0.87)	0.45 (0.64)	−0.06 (−0.06)	0.08 (0.30)	0.72 (0.76)	0.04 (0.12)	0.22 (0.29)	0.04 (−0.01)
		P1×P3	0.57 (0.70)	0.83 (0.89)	0.16 (0.34)	0.44 (0.44)	0.33 (0.66)	0.04 (0.13)	0.24 (0.31)	−0.12 (0.20)
		P1×P5	−0.19 (−0.08)	0.42 (0.44)	0.82 (0.84)	0.20 (0.29)	−0.16 (−0.15)	0.76 (0.81)	0.79 (0.89)	0.59 (0.71)
		P2×P3	0.11 (0.14)	0.38 (0.43)	0.02 (0.20)	0.83 (0.85)	−0.01 (0.00)	0.11 (0.18)	−0.11 (−0.13)	0.29 (0.35)
		P2×P4	0.65 (0.73)	0.62 (0.73)	−0.08 (−0.07)	−0.08 (0.10)	0.78 (0.84)	−0.03 (0.07)	0.29 (0.32)	−0.31 (−0.26)
		P2×P5	0.44 (0.66)	0.27 (0.75)	0.66 (0.76)	0.04 (0.45)	0.39 (0.55)	0.86 (0.95)	0.85 (0.92)	0.54 (0.66)
		P3×P5	0.32 (0.64)	0.30 (0.68)	0.60 (0.69)	−0.05 (0.13)	0.27 (0.66)	0.77 (0.89)	0.87 (0.94)	0.41 (0.57)
		P4×P5	0.11 (0.07)	0.14 (0.37)	0.63 (0.77)	0.25 (0.35)	−0.34 (−0.40)	0.77 (0.82)	0.74 (0.86)	0.74 (0.78)

			PS							
			P1×P2	P1×P3	P1×P5	P2×P3	P2×P4	P2×P5	P3×P5	P4×P5
PC	TS	P1×P2	0.71 (0.90)	0.59 (0.75)	0.04 (0.22)	0.36 (0.47)	0.68 (0.73)	0.28 (0.45)	0.52 (0.55)	−0.08 (0.00)
		P1×P3	0.55 (0.81)	0.83 (0.89)	0.09 (0.02)	0.26 (0.56)	0.71 (0.74)	0.12 (0.37)	0.47 (0.60)	0.15 (0.08)
		P1×P5	0.27	0.33	0.69	0.27	0.03	0.10	0.30	0.12
		P2×P3	0.27 (0.27)	0.20 (0.49)	0.07 (0.04)	0.77 (0.81)	0.21 (0.21)	0.07 (0.02)	0.49 (0.43)	0.00 (−0.05)
		P2×P4	0.35 (0.74)	0.56 (0.75)	0.23 (0.26)	0.19 (0.22)	0.86 (0.86)	0.06 (0.33)	0.41 (0.55)	−0.01 (0.11)
		P2×P5	0.42 (0.77)	0.09 (0.48)	0.19 (0.21)	0.04 (0.09)	0.39 (0.68)	0.76 (0.79)	0.50 (0.60)	0.03 (0.12)
		P3×P5	0.40 (0.73)	0.45 (0.81)	0.29 (0.36)	0.36 (0.62)	0.53 (0.75)	0.57 (0.56)	0.74 (0.76)	−0.12 (−0.21)
		P4×P5	−0.03	0.17	0.14	0.17	0.01	0.14	0.01	0.67

			PS							
			P1×P2	P1×P3	P1×P5	P2×P3	P2×P4	P2×P5	P3×P5	P4×P5
OC	TS	P1×P2	0.65 (0.83)	0.55 (0.73)	−0.09 (−0.06)	0.03 (0.15)	0.64 (0.68)	0.14 (0.29)	0.40 (0.48)	−0.04 (0.03)
		P1×P3	0.40 (0.68)	0.85 (0.91)	0.14 (0.04)	0.41 (0.71)	0.65 (0.68)	0.03 (0.21)	0.43 (0.56)	0.32 (0.19)
		P1×P5	0.17	0.22	0.71	0.17	−0.01	0.16	0.14	0.22
		P2×P3	0.16 (0.09)	0.24 (0.56)	0.05 (0.00)	0.79 (0.85)	0.27 (0.18)	0.08 (0.03)	0.35 (0.31)	0.16 (0.09)
		P2×P4	0.42 (0.70)	0.50 (0.72)	0.20 (0.22)	0.25 (0.29)	0.81 (0.81)	0.36 (0.41)	0.42 (0.51)	0.10 (0.08)
		P2×P5	0.15 (0.52)	−0.05 (0.33)	0.33 (0.35)	−0.11 (−0.08)	0.36 (0.60)	0.82 (0.83)	0.35 (0.48)	0.18 (0.27)
		P3×P5	0.37 (0.65)	0.47 (0.86)	0.27 (0.29)	0.48 (0.68)	0.48 (0.68)	0.52 (0.47)	0.67 (0.71)	−0.03 (−0.08)
		P4×P5	0.05	0.00	0.27	0.19	0.20	0.41	0.17	0.59

[a] SY, seed yield; TSW, thousand-seed weight; PY, protein yield; PH, plant height; PC, protein content; OC, oil content.
[b] TS, training set.
[c] PS, prediction set.

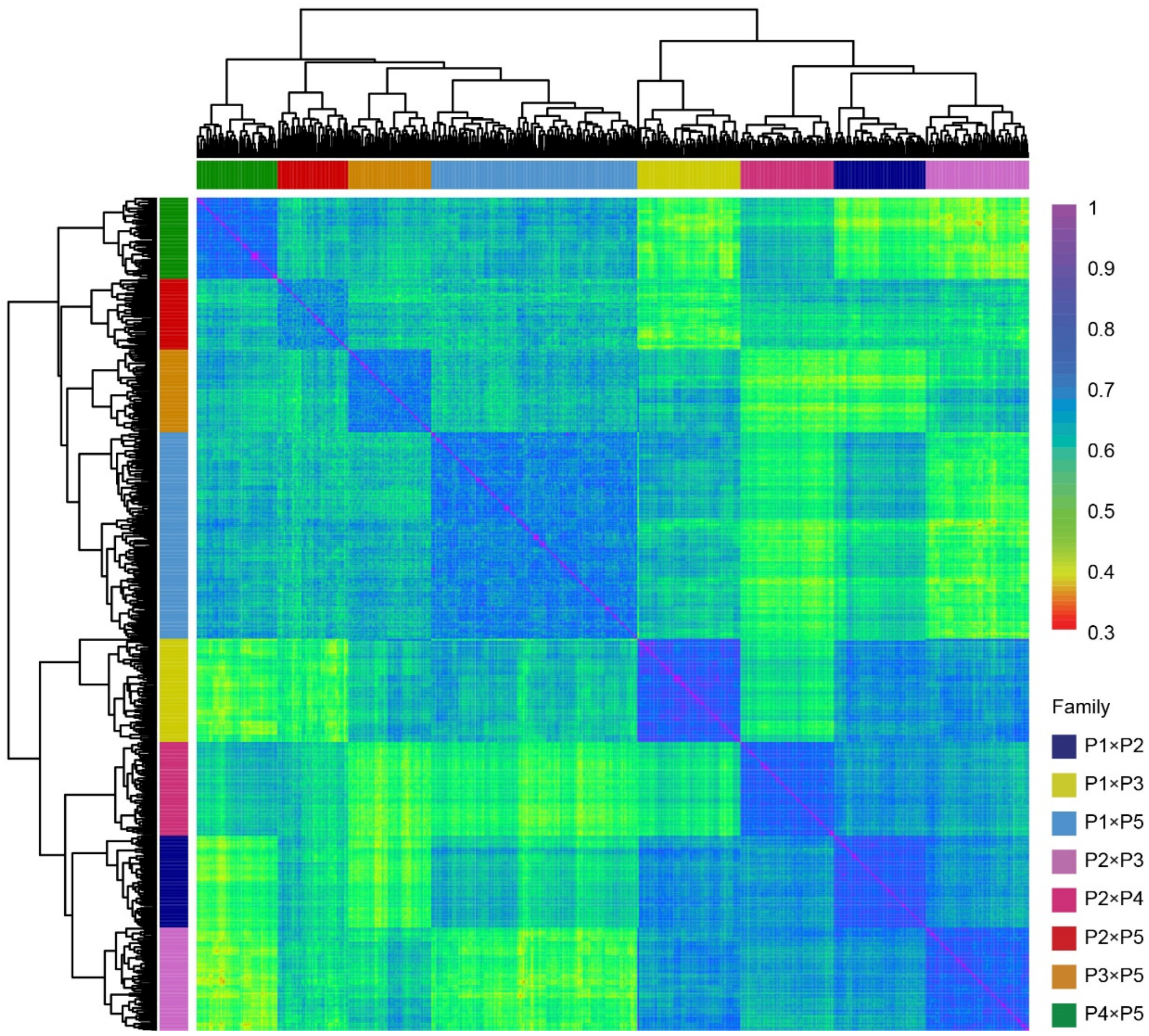

FIGURE S1 Genetic relatedness among the 944 RILs shown as kinship matrix calculated based on 10,893 markers.

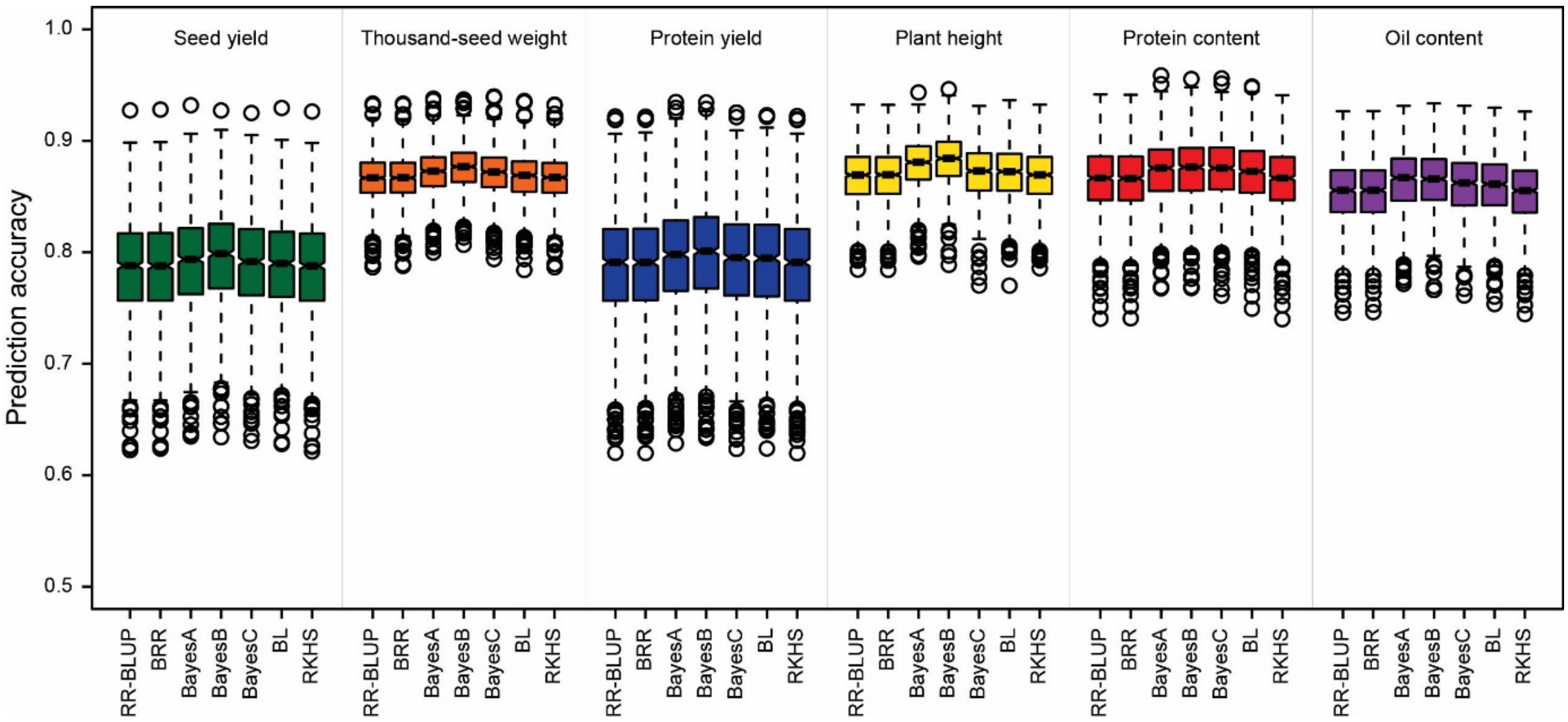

FIGURE S2 Comparison of the genomic prediction accuracy across all eight families assessed by fivefold cross-validation with different models. For Bayesian models, 20,000 iterations and 4,000 burn-in were used. RR-BLUP: ridge regression best linear unbiased prediction; BRR: Bayesian Ridge Regression; BL: Bayesian LASSO; RKHS: Bayesian Reproducing Kernel Hilbert Spaces Regression.

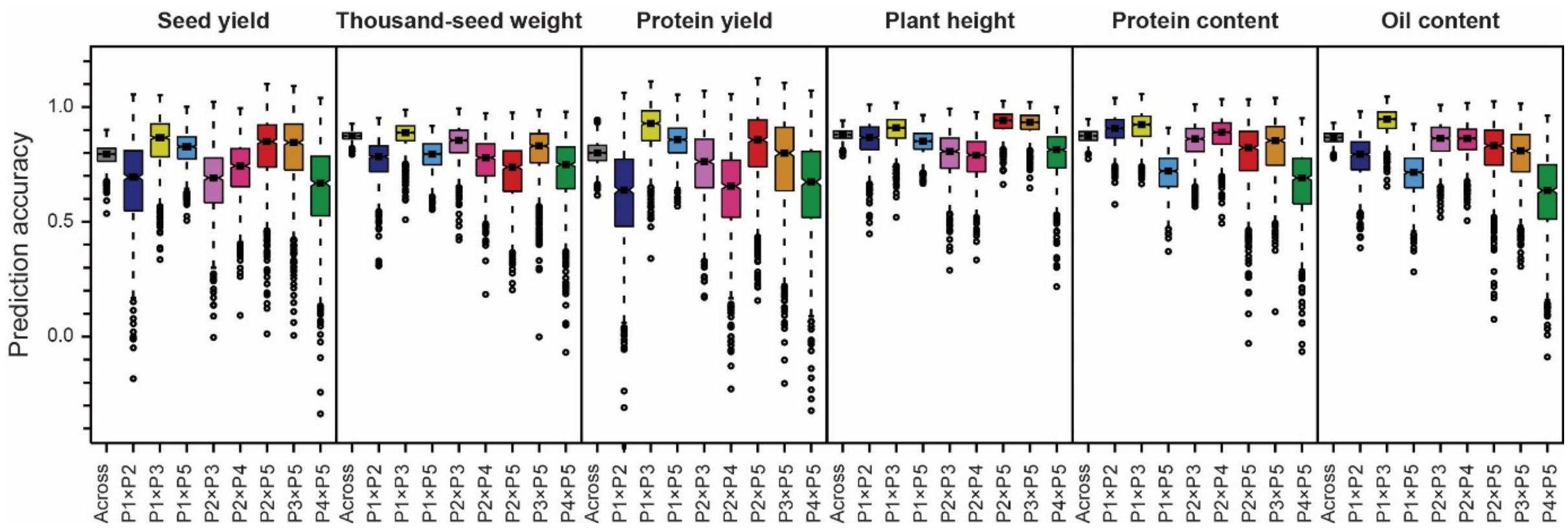

FIGURE S3 Prediction accuracy across families and for each of the eight families assessed by fivefold cross-validation employing a model with major QTL included as fixed effects and trained across all eight families.

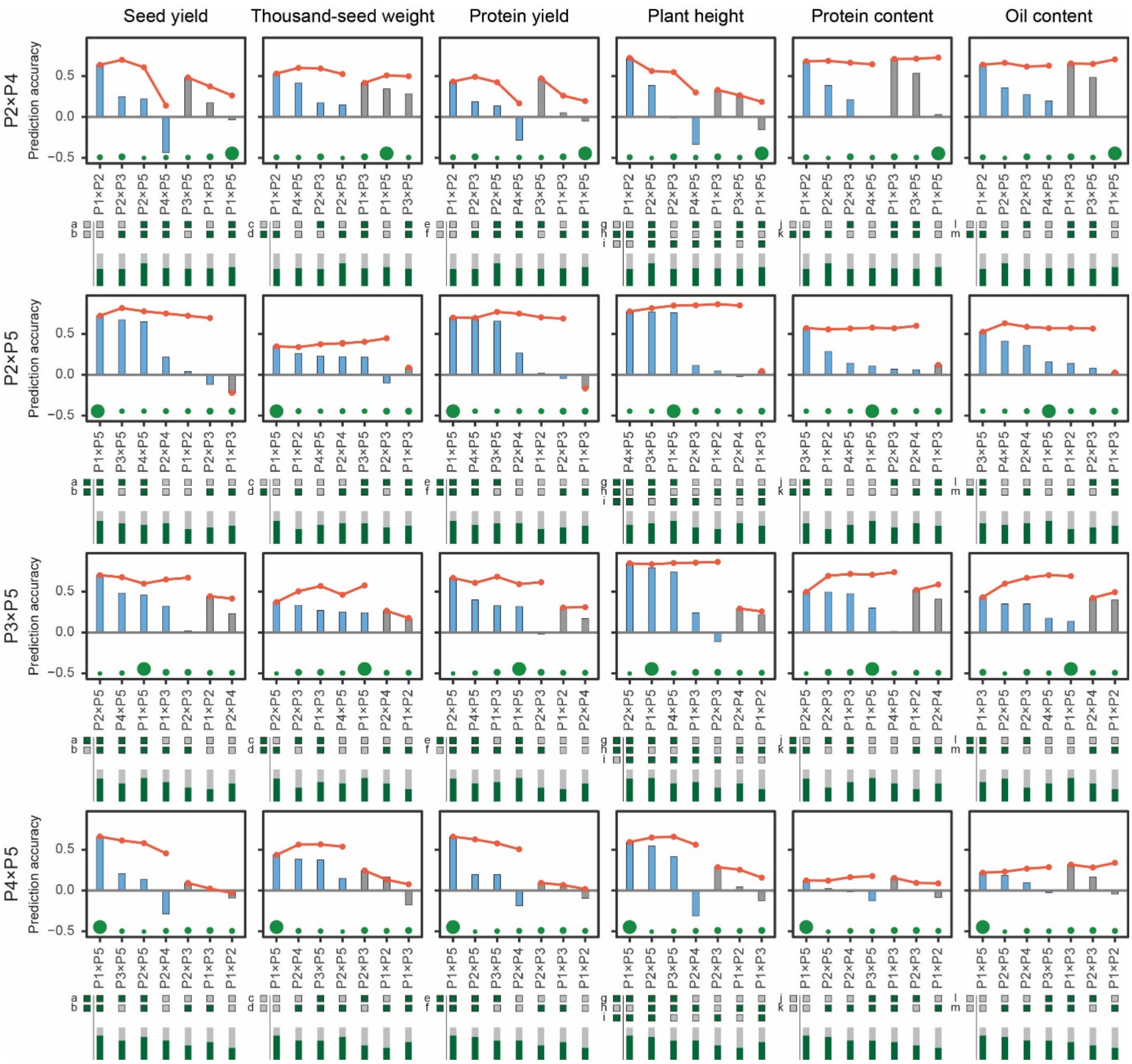

FIGURE S4 Genomic prediction accuracy of a single family based on single families or composite training sets. Results are shown for four families (P2×P4, P2×P5, P3×P5, P4×P5) that were predicted with single its half-sib families (Blue) or unrelated families (Grey), as well as composite training sets shown in red. For the latter, half-sib families and unrelated families were ranked in descending order of their prediction accuracy for the respective family and sequentially added to the training set. The green dots show the sample size of each family. The squares refer to the allelic state of the major QTL in each family, the two leftmost squares represent the family to be predicted, with green indicating segregation of the QTL. a, qSY1; b, qSY4; c, qTSW7; d, qTSW8; e, qPY2; f, qPY6; g, qPH2; h,qPH4; i, qPH5; j, qPC3; k, qPC5; l, qOC4; m, qOC5. The bar plots indicate the proportion of markers in that family for which an estimated effect is available for prediction (Green), i.e. the markers also segregating in the family used for prediction.

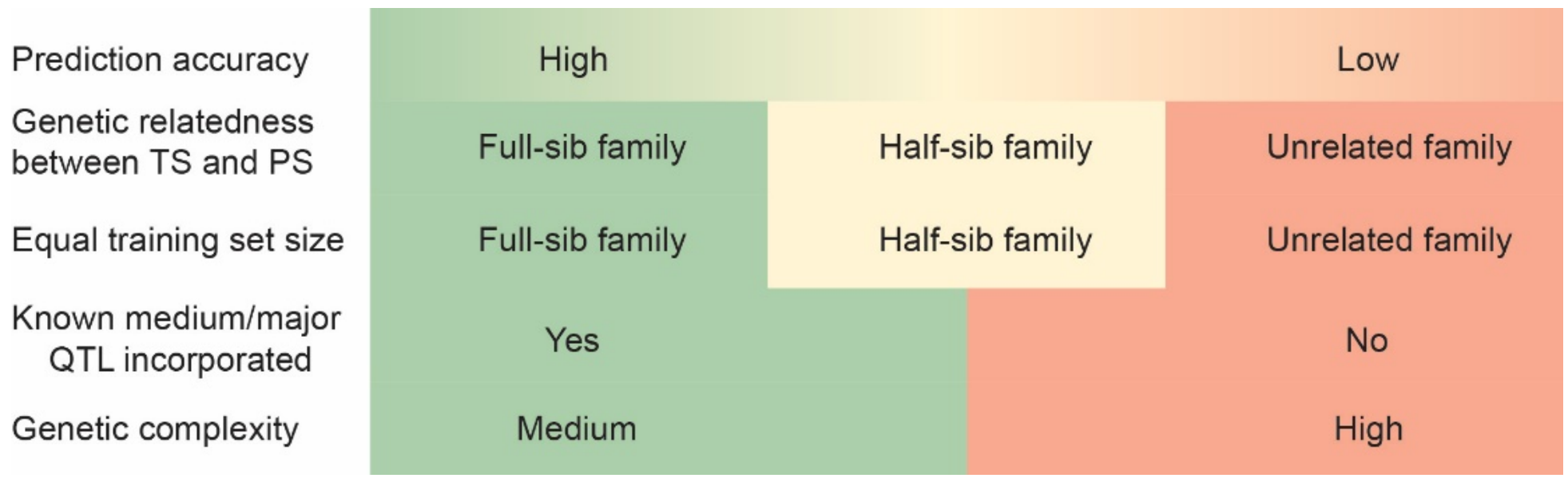

FIGURE S5 General guidelines on the optimization of genomic prediction in plant breeding. TS, training set; PS, prediction set.
